# Sealant Microleakage After Using Nano-Filled Bonding Agents on Saliva-Contaminated Enamel

**Published:** 2013-05

**Authors:** Mehrsa Paryab

**Affiliations:** ^1^Assistant Professor, Dental Research Center, Dentistry Research Institute, Department of Pediatric Dentistry, Faculty of Dentistry, Tehran University of Medical Sciences, Tehran, Iran

**Keywords:** Pit and fissure sealants; Saliva; Dental leakage; Dentin bonding agents; Nano-filled

## Abstract

**Objective:** The efficacy of correctly applied fissure sealants has been revealed in the prevention of caries. Saliva and moisture contamination of the etched enamel surface before sealant placement can decrease the bonding strength of the sealant to the enamel. The aim of this study was to test the new bonding agents containing nano-fillers in order to reduce the negative effect of saliva contamination on the sealant micro leakage.

**Materials and Methods:** Seventy five sound human premolars were randomly assigned to five equal groups as follows: **Group A:** etching, sealant; **Group B:** etching, saliva contamination, sealant; **Group C:** etching, saliva contamination, Single bond, sealant; **Group D:** etching, saliva contamination, Adper Single bond 2, sealant; **Group E:** etching, saliva contamination, N Bond, sealant. The samples were thermo-cycled and immersed in basic fuchsine 0.5% by weight. Then, the teeth were sectioned bucco-lingually and parallel to the long axis into two segments. Finally, the length of dye penetration at the sealant-tooth interface was scored according to a four-point scale.

**Results:** Micro-leakage was higher in group B compared to the other groups, while there were no differences among the evaluated dentin adhesives.

**Conclusion:** The use of nano-filled bonding agents as an intermediate layer between the etched enamel and the sealant can reduce sealant micro-leakage after saliva contamination at the level of the uncontaminated enamel.

## Introduction

Dental caries is one of the most common challenges in childhood [1]. Several attempts have been made to reduce the occurrence of dental caries. Sealing the pits and fissures in occlusal surfaces of the posterior teeth is an effective preventive procedure to reduce dental caries [1, 2]. 

The retention rate and optimal sealing along the enamel-sealant interface are key factors for success of this type of treatment. The main reason for sealants failure is contamination of the etched enamel surfaces with saliva before the sealant placement. Exposure to saliva causes surface coating over the etched enamel surfaces which by itself prevents resin penetration into their porosity [3]. The overall bonding strength to saliva-contaminated enamel and marginal sealing is markedly lower than non-contaminated ones [4-14]. 

Providing isolated and dry field is usually very difficult in young children, patients with special needs and patients who have newly erupted teeth. In these situations, postponing the sealing procedure to the future or repeating the etching process is preferred [15]. Re-etching alters the microstructure of the tooth enamel [6]. As an alternative procedure, the use of hydrophilic bonding agents prior to sealant placement improves the bond strength and reduces micro-leakage of the sealant at the level of uncontaminated enamel [5, 9-14]. Some other studies do not confirm the positive effect of the bonding agent [16].

In the past two decades, in the dentin bonding system, considerable advancement has been made. In the new agents, the addition of nano-fillers with an average particle size of 12 nm increases the penetration of resin monomers and the hybrid layer thickness, which in turn improves the mechanical properties of the bonding system [17- 22].

The sealing ability of the bonding system in the margins of the restorations needs further research in different settings. The aim of this study was to test the effect of new bonding agents containing nano-fillers on reducing the negative impact of saliva contamination of the sealant micro-leakage for the first time. 

## MATERIALS AND METHODS

Seventy five sound human premolars, extracted for orthodontic purposes within a 6-month period, were selected and disinfected with thymol 0.2%. The teeth were cleaned with water/pumice slurry and were randomly assigned to five equal groups (N=15). The occlusal pits and fissures of the teeth were etched with 37% phosphoric acid (Ultra Etch, Ultra dent product INC, USA) for 15 seconds; then they were rinsed thoroughly for 5 seconds and dried with oil-free compressed air to obtain a uniformly whitish, dull, chalk like appearance. Bonding procedures in each group were performed as follows: 

**Group A:** Fissure sealant (Concise, 3M ESPE, USA) was applied into etched pits and fissures including 2 to 3 mm cuspal incline and cured for 20 seconds with a power halogen light-curing unit (Coltolux 75, Colten, USA) with a 450 mw/mm² output.

**Group B:** A thin layer of artificial saliva (Potassium: 20 meq.lit, Chloride: 27.4 meq.lit, Sodium: 0.22 mg, Magnesium: 0. 1.5 mg, Calcium: 0.6 mg, Fluoride: 0.5 mg, Phosphorous: 0.21 mg, Hydroxy propyl methyl cellulose 3%) was brushed onto the enamel so that the whole surface area of the enamel was covered. This was left undisturbed for 10 seconds prior to drying. The surface was then gently dried for 20 seconds, and finally the pits and fissures were sealed.

**Group C:** Contamination with saliva was performed as in group B. Two consecutive coats of Single Bond (3M, EPSE, USA) were applied to the etched enamel for 15 seconds with gentle agitation using a fully-saturated applicator. The surface was then gently dried for 15 seconds to evaporate solvents. Adhesive was light cured for 10 seconds, and finally the pits and fissures were sealed. 

**Group D:** Contamination with saliva was performed as in group B. Before the sealant placement, two layers of Adper Single Bond 2 (3M, EPSE, USA) containing colloidal filler 5nm in size and 10% by weight were applied, dried for 15 seconds, and then light cured for 10 seconds.

**Group E:** After the etching process and contamination with saliva, a thick layer of Tetric N-bond (Ivoclar Vivadent, Liechtenstein) containing Sio_2_ fillers was applied on the enamel surface and brushed in for at least 10 seconds. Excess material and the solvent were removed by a gentle stream of air. According to the manufacturer’s instructions, the adhesive was light cured for 10 seconds, and then the pits and fissures were sealed.

According to the ISO/TR 11405 standardization [23], the samples were stored for at least 24 hours before testing in distilled water at 37°C and thermo-cycled for 500 cycles between 5 -55, with a 50 s dwell time. Following thermo-cycling, the teeth were sealed at the root apices and coated with two layers of nail polish, except for the restorations and 1 mm rim of tooth structure around the restoration. The samples were immersed in basic fuchsine 0.5% by weight (Merk, Germany) for 24 hours and then thoroughly rinsed in tap water. Then, In order to prevent chipping of the material, the teeth were embedded in self curing acrylic resin (Marlic Med Co, Iran). The prepared blocks were cut parallel to the long axis into mesial and distal fragments. 

The length of dye penetration at the sealant-tooth interface was examined under a light microscope with X 40 magnification. 

The criterion for the amount of dye micro-leakage was the maximum level of dye penetration. Scoring was based on the following criteria:

**Grade 0:** no penetration; **Grade 1:** dye penetration up to one-third of the sealant-tooth interface; **Grade 2:** dye penetration extending from one-third to two-thirds of the length of the sealant- tooth interface; **Grade 3:** dye penetration more than two-thirds of the length of the sealant-tooth interface.

The data were analyzed using Kruskal Wallis and pair-wise comparison tests. All statistical tests were set at a significant level of p < 0.05. P-values for pairwise comparisons were adjusted by related options in IBM SPSS Statistics version 20.

## RESULT

A total of 150 sections were examined for micro-leakage. The percentage of each score for all groups has been shown in Table 1. 

**Table 1:** Micro-Leakage Grades in Experimental Groups

	**Grade 0**	**Grade 1**	**Grade 2**	**Grade 3**
**Group A**	23(77%)	6(20%)	1(3%)	0(0%)
**Group B**	15(50%)	3(10%)	3(10%)	9(30%)
**Group C**	28(93%)	2(7%)	0(0%)	0(0%)
**Group D**	25(83%)	5(17%)	0(0%)	0(0%)
**Group E**	27(90%)	1(3%)	2(7%)	0(0%)

**Group A:** etching, sealant

**Group B:** etching, saliva contamination, sealant

**Group C:** etching, saliva contamination, Single bond, sealant

**Group D:** etching, saliva contamination, Single bond 2, sealant

**Group E:** etching, saliva contamination, N Bond, sealant

Statistical analysis results showed a significant difference in the overall micro-leakage between the groups (p< 0.05).

Results of the pairwise statistical tests showed a higher micro-leakage in group B in comparison to the other groups (p< 0.05). There were no differences among the evaluated bonding agents (p>0.05) (Table 2).

## Discussion

It is generally accepted that adequate isolation is the most critical aspect of the sealant application process. Re-establishing the etched enamel surface morphology after salivary contamination can be provided through washing, re-etching and adding an adhesive layer on the contaminated and previously etched enamel prior to sealant placement. The aim of this study was to test the new bonding agents containing nano-fillers to prevent micro-leakage in saliva-contaminated enamel.

Studies using two bottles and total etch dentin adhesive systems have shown good results in reducing the effects of moisture on sealant retention. 

Hydrophilic monomers and acetone-based solvents can displace the saliva, and penetrate to the porosities in these systems; consequently improving the bonding of sealants to saliva-contaminated enamel in some specific situations [5, 10-12].

Sixth and seventh generations of dentin bonding were also studied [13, 14]. Bassir et al. (2009) compared two self-etch bonding agents, protect Bond and S_3_ Bond with total etch systems. The results showed that self-etch bonding agents can not reduce sealant micro-leakage after saliva contamination at the level of the uncontaminated enamel and are therefore not advised [13]. 

Karami et al. (2010) showed that using Adhes and I Bond systems decrease the sealant microleakage in the saliva contaminated group. These new agents from self-etch generations have an acidic hydrophilic monomer and can be easily used on the etched enamel after contamination with saliva or moisture [14].

In the new nano-filled agents, by adding minute size fillers to the adhesives, the elastic modulus increases. 

**Table 2:** Pairwise Comparison Analysis Test

**Groups**	**P- value** **(Sig- Adjusted)**	**Groups**	**P- value** **(Sig- Adjusted)**
**Groups A & B**	**0.002(0.023)**	**Groups B & D**	**0.00(0.003)**
**Groups A & C**	**0.147(1.00)**	**Groups B & E**	**0.00(0.00)**
**Groups A & D**	**0.543(1.00)**	**Groups C & D**	**0.400(1.00)**
**Groups A & E**	**0.283(1.00)**	**Groups C & E**	**0.707(1.00)**
**Groups B & C**	**0.00(0.00)**	**Groups D & E**	**0.642(1.00)**

**Group A:** etching, sealant

**Group B:** etching, saliva contamination, sealant

**Group C:** etching, saliva contamination, Single bond, sealant

**Group D:** etching, saliva contamination, Single bond 2, sealant

**Group E:** etching, saliva contamination, N Bond, sealant

Based on the manufacturer, nano-particles acting as cross links will reduce the dimensional changes [19, 20]. The type of nano-fillers and the method that these particles are incorporated affect the adhesive viscosity and penetration ability of the resin monomers into collagenic fibers spaces [20- 22]. Nano-fillers, with dimensions larger than 15-20 nm or a content of more than 1.0 percent by weight, both can increase the viscosity of the adhesives, and may cause accumulation of the fillers over the top of the moistured surface. These clusters can act as flaws which may induce cracks and cause a decrease in the bond strength [18, 20]. Therefore, concerns about the sealing ability of these bonding agents on saliva contaminated surfaces increase. 

The results of the present investigation indicated that application of nano-filled bonding agents on the saliva-contaminated enamel can reduce the sealant micro-leakage to the same level observed in the control group where no contamination was performed. The efficiency of N Bond and Adper Single Bond 2 are contributed to hydrophilic monomers and also the proper content of nano-fillers [23]. The hydrophilic monomers with a high diffusion rate in alcohol-based solvents are able to remove any residual moisture from the etched enamel and enhance resin penetration. 

It seems that the size and weight of colloidal nano-fillers in N-bond and Adper Single Bond 2 are sufficient. So; in a situation in which the moisture or saliva contaminate the etched enamel, the fillers do not form agglomerated clusters; and can penetrate into the enamel porosities along with the monomers of the bonding agent. This ability can overcome the many negative aspects of contamination by saliva as well as non-filled bonding agents. It is suggested to evaluate the shear bond strength of the sealant to the enamel after using the nano-filled adhesive agents on the saliva-contaminated enamel.

## CONCLUSION

In a situation, in which control of saliva and isolation is impossible, the use of nano-filled bonding agents as an intermediate layer between the etched enamel and the sealant can be efficient in reducing sealant micro-leakage after saliva contamination at the level of the uncontaminated enamel.

## References

[1] McDonald RE, Avery DR, Stookey GK, Chin JR, Kowolik JE, Dean JA, Avery DR, McDonald RE (2011 ). Dental caries in the child and adolescent. Dentistry for the child and adolescent.

[2] Hicks J, Flaitz CM, Pinkham JR, Casa Massimo PS, McTigue DJ, Fields HW, Nowak AJ (2005). pit and fissure sealants and conservative adhesive restorations: Scientific and Clinical Rationals. Paediatric dentistry -infancy through adolescence.

[3] Hormati AA, Fuller JL, Denehy GE (1980). Effects of contamination and mechanical disturbance on the quality of acid- etched enamel. J Am Dent Assoc..

[4] Thomson JL, Main C, Gillespie FC, Stephen KW (1981). The effect of salivary contamination on fissure sealant – enamel bond strength. J Oral Rehabil..

[5] Hitt JC, Feigal RJ (1992). Use of a bonding agent to reduce sealant sensitivity to moisture contamination: an in vitro study. Pediatr Dent..

[6] Correr GM, Caldo-Teixeira AS, Alonso RC, Puppin-Rontani RM, Sinhoreto MA, Correr- Sobrinho L (2004). Effect of salava contamination and re-etching time on the shear bond strength of a pit and fissure sealant. J Appl Oral Sci..

[7] Barroso JM, Torres CP, Lessa FC, Pecora JD, Palma- Dibb RG, Borsatto MC (2005). Shear bond strength of pit-and-fissure sealant to saliva-contaminated and non-contaminated enamel. J Dent Child..

[8] Lepri TP, Souza-Gabriel AE, Atoui JA, Palma- Dibb RG, Pecora JD, Milori Corona SA (2008). Shear bond strength of a sealant to contaminated- enamel surface: influence of erbium: yttrium- aluminum- garnet laser pretreatment. J Esthet Restor Dent..

[9] Feigal RJ, Hitt J, Splieth C (1993). Retaining sealant on salivary contaminated enamel. J Am Dent Assoc..

[10] Hebling J, Feigal RJ (2000). Use of one-bottle adhesive as an intermediate bonding layer to reduce sealant microleakage on saliva-contaminated enamel. Am J Dent..

[11] Duangthip D, Lussi A (2003). Microleakage and penetration ability of resin sealant versus bonding system when applied following contamination. Pediatr Dent..

[12] Askarizadeh N, Norouzi N, Nemati S (2008). The effect of bonding agents on the microleakage of sealant following contamination with saliva. J Indian Soc Pedod Prev Dent..

[13] Basir L, Khanehmasjedi M, Kaviani A, Haghighizadeh M, Khalili E (2009). An in vitro comparison of microleakage of two self- etch adhesive and the one- bottle adhesive used in pit and fissure sealant with or without saliva contamination. J Dent Sch..

[14] Karami Nogourani M, Javadi Nejad Sh, Homayunzadeh M (2010). Sealant Microleakage in Saliva-Contaminated Enamel: Comparison between three adhesive systems. J Dent Sch..

[15] Roberson TM, Roberson TM, Heymann HO, Edward J, Swift Jr (2006). Cariology: the lesion, etiology, prevention and control. Art and science of operative dentistry.

[16] Torres CP, Balbo P, Gomes-Silva JM, Ramos RP, Palma-Dibb RG, Borsatto MC (2005). Effect of individual or simultaneous curing on sealant bond strength. J Dent Child (Chic).

[17] Sutalo J, Knezevic A, Tarle Z (2001). Influence of dentine adhesive with Nanofiller on post restorative Sensitivity. Acta Stomatol Croat..

[18] Kim JS, Cho BH, Lee IB, Um CM, Lim BS, Oh MH (2005). Effect of the hydrophilic nanofiller loading on the mechanical properties and the microtensile bond strength of an ethanol-based one-bottle dentin adhesive. J Biomed Mater Res B Appl Biomater..

[19] Başaran G, Ozer T, Devecioğlu Kama J (2009). Comparison of a recently developed nanofiller self-etching primer adhesive with other self-etching primers and conventional acid etching. Eur J Orthod..

[20] Kasraei SH, Atai M, Khamverdi Z, Khalegh Nejad S (2009). Effect of nanofiller addition to an experimental dentin adhesive on microtensile bond strength to human dentin. J Dent (Tehran)..

[21] Miyazaki M, Ando S, Hinoura K, Onose H, Moore BK (1995). Influence of filler addition to bonding agents on shear bond strength to bovin dentin. Dent Mater..

[22] Nunes MF, Swift EJ, Perdigao J (2001). Effects of adhesive composition on microtensile bond strength to human dentin. Am J Dent..

[23] Siadat H, Mirfazaelian A (2002). Microleakage and its measurement methods. J Dent Med..

